# Influence of location-dependent sex difference on PD-L1, MMR/MSI, and EGFR in colorectal carcinogenesis

**DOI:** 10.1371/journal.pone.0282017

**Published:** 2023-02-21

**Authors:** Jina Choi, Nayoung Kim, Ryoung Hee Nam, Jin Won Kim, Chin-Hee Song, Hee Young Na, Gyeong Hoon Kang

**Affiliations:** 1 Department of Internal Medicine, Seongnam, Korea; 2 Seoul National University College of Medicine, Seoul, Korea; 3 Department of Internal Medicine and Liver Research Institute, Seoul, Korea; 4 Department of Pathology, Seoul National University Bundang Hospital, Seongnam, Korea; 5 Department of Pathology, Seoul National University College of Medicine, Seoul, Korea; CNR, ITALY

## Abstract

**Background:**

The incidence and mortality rates of colorectal cancer (CRC) has been reported to be strongly associated to sex/gender difference. CRC shows sexual dimorphism, and sex hormones have been shown to affect the tumor immune microenvironment. This study aimed to investigate location-dependent sex differences in tumorigenic molecular characteristics in patients with colorectal tumors, including adenoma and CRC.

**Methods:**

A total of 231 participants, including 138 patients with CRC, 55 patients with colorectal adenoma, and 38 healthy controls, were recruited between 2015 and 2021 at Seoul National University Bundang Hospital. All patients underwent colonoscopy and acquired tumor lesion samples were further analyzed for programmed death-ligand 1 (PD-L1), epidermal growth factor receptor (EGFR) expression, deficient mismatch repair (dMMR), and microsatellite instability (MSI) status. This study was registered with ClinicalTrial.gov, number NCT05638542.

**Results:**

The average of combined positive score (CPS) was higher in serrated lesions and polyps (lesions/polyps) compared to conventional adenomas (5.73 and 1.41, respectively, *P* < 0.001). No significant correlation was found between sex and PD-L1 expression within the groups, regardless of histopathological diagnosis. In multivariate analysis where each sex was further stratified by tumor location due to their interaction in CRC, PD-L1 expression was inversely correlated with males having proximal CRC with a CPS cutoff of 1 (Odds ratio (OR) 0.28, *P* = 0.034). Females with proximal CRC showed a significant association with dMMR/MSI-high (OR 14.93, *P* = 0.032) and high EGFR expression (OR 4.17, *P* = 0.017).

**Conclusion:**

Sex and tumor location influenced molecular features such as PD-L1, MMR/MSI status and EGFR expression in CRC, suggesting a possible underlying mechanism of sex-specific colorectal carcinogenesis.

## Introduction

Colorectal cancer (CRC), the second leading cause of cancer death worldwide [1], is associated with higher mortality and age-standardized incidence rates in males compared to females across different regions of the world [2]. The divergence in genetic and pathophysiological factors between males and females indicates sex differences, while gender differences refer to behavioral and modifiable risk factors in CRC, such as dietary habits, body mass index (BMI), alcohol consumption, smoking, and physical activities [3]. In fact, female patients with CRC at pre-menopausal age exhibit survival benefits compared to age-matched male or older female patients over 50 years of age [1]. In contrast, male patients with CRC over 65 years of age were reported to have a better survival outcome compared to females of the same age [4]. These differences appear to originate partially from sex steroid hormones, mainly estrogen, which play a protective role in colorectal carcinogenesis [1,2,5] as well as the development of colorectal adenomas [6]. Meanwhile, testosterone strongly enhances azoxymethane/dextran sulfate sodium-induced colorectal cancer development in C57BL/6 mice [7].

Accumulating evidence supports that immune system also shows substantial sex differences [2,8]. In recent years, immune checkpoint inhibitors (ICI) targeting the programmed death 1 receptor (PD-1) or its ligand (PD-L1) have changed the landscape of systemic cancer treatment [9]. PD-L1 expression in tumors and/or infiltrating immune cells attenuates T-cell activation by interacting with PD-1 on immune cells, which enables immune evasion of tumor cells [10]. Besides, PD-L1 has been shown to exert immune-independent tumorigenicity on a variety of tumor cell types [11], and interact with key molecules in tumor progression such as EGFR [12]. However, few studies have investigated the role of immune environment in both colorectal adenomas and carcinomas in terms of carcinogenesis [13,14]. In addition, no studies have examined the expression of PD-L1 in colorectal adenomas including conventional adenomas and serrated precursor lesions, and its association with sex and tumor location.

Females show a higher frequency of right-sided (proximal) CRCs than males, suggesting that sex differences exist at multiple levels in CRC [2,15]. Furthermore, tumor location is known to be associated with differences in key tumorigenic molecular features, including microsatellite instability (MSI) and epidermal growth factor receptor (EGFR) [16,17]. Current medical guidelines for colorectal cancers, including National Comprehensive Cancer Network and European Society for Medical Oncology have recently added primary tumor location for cetuximab and panitumumab, anti-EGFR antibodies, in their recommendations for making therapeutic decisions [18,19]. Likewise, further investigations have been made to combined ICIs with target agents or conventional chemotherapies, in efforts to increase the efficacy of ICIs in CRC [20]. However, prognostic discrepancies in immunotherapy have been reported among cancers including CRC depending on the sex [21,22].

Based on this background, we hypothesized that tumorigenic molecular markers could differ depending on sex and tumor location, which might affect the therapeutic approach of CRC. Therefore, the aim of this study was to evaluate the influence of location-dependent sex/gender differences in colorectal adenoma and CRC and to investigate the sex- and gender-associated characteristics of molecular markers, including PD-L1, mismatch repair (MMR)/MSI status and EGFR.

## Materials and methods

### Study participants

**Fig 1:**
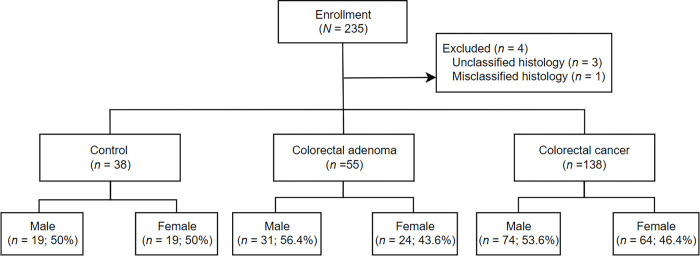
Study flow chart of the enrolled participants and further classification according to sex.

Study participants who visited Seoul National University Bundang Hospital from March 2015 to January 2021 for regular check-ups for surveillance of CRC or due to gastrointestinal symptoms such as abdominal discomfort, diarrhea, and constipation were prospectively enrolled. Patients were recruited based on the following inclusion criteria: (a) histologically confirmed colorectal adenocarcinoma or (b) colorectal adenomas greater than or equal to 10 mm in diameter according to the endoscopic presentation. The controls were defined as the participants who had a single erosion or erythema without evidence of CRC or adenoma, which was confirmed by histology and endoscopic presentation, and they agreed to participate in this study. The following patients were excluded: (a) history of colorectal cancer, polyps or colectomy before the first surveillance colonoscopy, (b) hereditary CRC syndromes such as familial adenomatous polyposis or lynch syndrome, (c) a family history of CRC or colorectal polyps in at least one first-degree relative, (d) inflammatory bowel disease, and (e) incomplete colonoscopy or incomplete clinical information. Finally, 231 participants, including 38 healthy controls, 138 patients with CRC, and 55 patients with colorectal adenoma were selected, which were further categorized into two groups according to sex. (Fig 1). The data were collected from both the participant questionnaire and medical records and included sex, age, BMI, and social history such as alcohol consumption and smoking. This study was reviewed and approved by the Institutional Review Board of SNUBH (IRB No. B-1305/203-009), and written, informed consent was obtained from all participants. All the investigations were conducted in accordance with the ethical guidelines of the Declaration of Helsinki (1898). This study was registered with ClinicalTrial.gov, number NCT05638542.

### Endoscopic and histologic analysis

All patients were examined using video colonoscopes (Olympus CF-240I; Olympus, Tokyo, Japan) by an experienced (over 30 years) gastroenterologist (N. K.). The locations of the biopsy lesions were classified as follows: the proximal colon (cecum, ascending colon, hepatic flexure, and transverse colon) and distal colon (splenic flexure of the colon, descending colon, sigmoid, and rectum). Histologic diagnosis of the specimens was evaluated by a Gastroenterology pathologist (H. Y. N.) based on the 2019 WHO guidelines for digestive system tumors [23]. Colorectal adenomas were categorized as conventional adenomas, including tubular, tubulovillous and villous adenomas, or serrated lesions and polyps (lesions/polyps), including sessile serrated lesions (SSL), traditional serrated adenomas (TSA), and serrated adenomas, unclassified.

### Immunohistochemistry for PD-L1

Immunohistochemistry (IHC) for PD-L1 using 22C3 pharmDx antibody (mouse monoclonal, 22C3, Dako, Carpinteria, CA, USA) was performed on the Autostainer Link 48 with EnVision DAB Detection System (Agilent Technologies, Santa Clara, CA, USA) according to the manufacturer’s recommendations [24].

**Fig 2:**
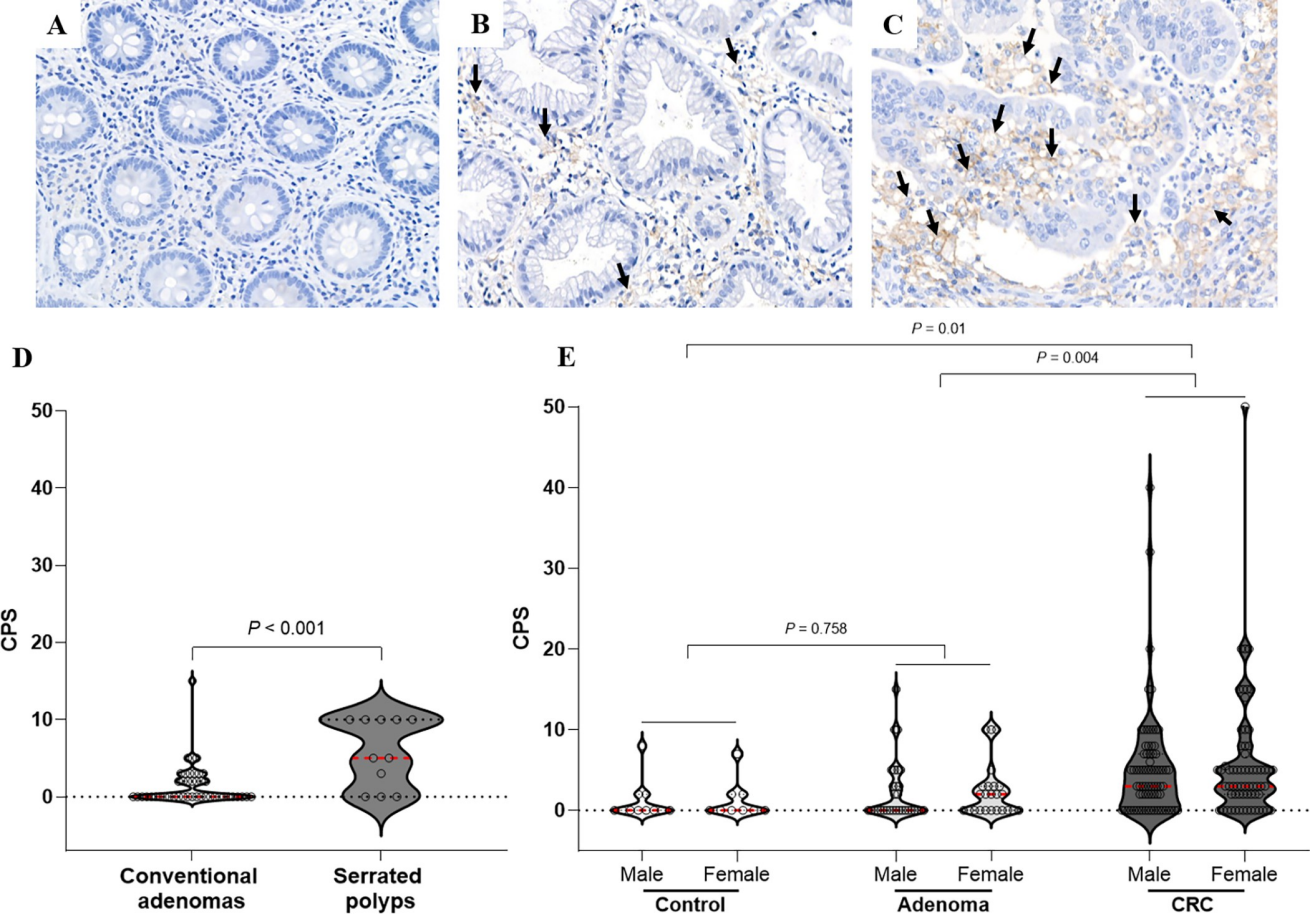
Expression of PD-L1 in colorectal tissues depending on histology. Representative immunohistochemical results of PD-L1 expression (arrows): (A) negative normal control, (B) sessile serrated lesion (adenoma), and (C) CRC (magnification, x20). (D) A statistically significant increase in PD-L1 combined positive score (CPS) was observed in serrated lesions/polyps within colorectal adenomas. (E) Colorectal cancer (CRC) showed a significantly higher PD-L1 CPS than control and colorectal adenomas.

PD-L1 expression in the membrane of tumor cells, and membrane and/or cytoplasm of tumor-associated immune cells, macrophages and lymphocytes, was scored from stained slides (Fig 2A–2C). The combined positive score (CPS) was calculated and defined as the total number of PD-L1 positive cells including tumor and mononuclear inflammatory cells, divided by the number of all viable tumor cells in colorectal adenomas and CRCs, followed by multiplication with 100 [25]. The expression of PD-L1 in normal colonic mucosa was defined by its expression in the membrane of epithelial cells and the membrane and/or cytoplasm of macrophages or lymphocytes in the mucosa. CPS was defined as the total number of PD-L1-positive mucosal epithelial cells and mucosal mononuclear immune cells divided by the number of mucosal epithelial cells.

### IHC of EGFR and MMR

The expression levels of EGFR (rabbit monoclonal, prediluted; Ventana Medical Systems, Oro Valley, AZ, USA) and the status of four MMR proteins including MLH1 (mouse monoclonal, prediluted; Roche,Indianapolis, IN, USA), MSH2 (mouse monoclonal, 1:200; Cell Marque,Rocklin, CA, USA), MSH6 (mouse monoclonal, 1:100; Cell Marque), and PMS2 (rabbit monoclonal, prediluted; Roche) were analyzed by immunohistochemistry, according to the manufacturer’s instructions.

To evaluate the overexpression of EGFR in CRC, strong or moderate intensity of complete staining in ≥ 10% of tumor cells was scored as 3+ or 2+, respectively, while cases with < 10% of tumor cells, including those expressing weak and incomplete staining, were scored as 1+. No staining or non-specific staining of tumor cells was coded as 0. Scores of 0 and 1+ were defined as EGFR low, whereas scores of 2+ and 3+ were regarded as EGFR high (S1A and S1F Fig), as previously reported [26–28].

The expression of each MMR protein (MLH1, MSH2, MSH6, and PMS2) was considered negative when nuclear staining in tumor cells was completely absent. Adjacent normal epithelial cells or lymphocytes were used as internal controls. Intact nuclear staining of all four proteins in CRC was classified as proficient MMR (S1B–S1E Fig), whereas negative staining of one or more of the four MMR proteins was defined as dMMR (S1G–S1J Fig) [29]. All specimens were analyzed and reviewed by a pathologist (H.Y. N) without the knowledge of clinical information.

### Polymerase chain reaction for MSI

Polymerase chain reaction (PCR) was performed to analyze MSI status of tumor cells using five National Cancer Institute (NCI) markers (BAT-26, BAT-25, D5S346, D17S250, and S2S123) in CRC. Using PCR products, the MSI status was determined using an automated DNA sequence analyzer (ABI 3731 Genetic Analyzer, Applied Biosystems, Foster City, CA, USA) by assessing the alteration of the allele profiles in tumor cells compared with matched non-neoplastic normal tissues. Samples were denoted as MSI-high if two or more NCI markers showed instability, MSI-low if one marker was unstable, or microsatellite stable (MSS) if no instability was present [30].

### Statistical analysis

Continuous and categorical variables were compared using Student’s *t*-test or Mann−Whitney *U* test and Pearson’s chi-square test or Fisher’s exact test, respectively. Simple logistic regression was used to evaluate the factors associated with sex differences or CPS levels. Multivariate multiple regression analysis was performed to identify independent predictive factors of putative molecular markers. Kaplan-Meier curves and log-rank test were used for survival analysis. All results were considered statistically significant at a *p*‐value of less than 0.05. A minimal target sample size of 114 participants with CRC was calculated considering 13% of MSI-high incidence in CRC, as previously reported [31], and the minimum of 40 participants with colorectal adenomas was required based on the prevalence of MSI in serrated lesions/polyps, 53%, and tubular adenomas, 13%, from the previous report [32], with a power of 80% and a type I error rate of 5%. All statistical analyses were performed using SPSS 28.0 (SPSS, Chicago, IL, USA), and graphs were generated using GraphPad Prism 9.0 (GraphPad Software, San Diego, USA). The Division of Statistics in Medical Research Collaborating Center at Seoul National University Bundang Hospital assisted with statistical analysis.

## Results

### Baseline characteristics of study participants by tumor diagnosis and sex

A total of 231 participants, including 138 patients with CRC, 55 patients with adenoma and 38 healthy controls were recruited (Fig 1); mean age was 63.4 years (range: 22–93 years), including 107 females (46.3%) and 124 males (53.7%). Baseline clinical characteristics varied across the groups, although no statistical significance was found for most variables (Table 1). Among the groups, the frequency of individuals aged 65 years or older was the highest in the patients with CRC (60.9%).

**Table 1:** Baseline characteristics of the study cohort in control, colorectal adenoma, and colorectal carcinoma.

	Control, *n* (%)				Colorectal adenoma, *n* (%)				Colorectal carcinoma, *n* (%)				*P*-val.^2^
	Total	Male	Female	*P*-val.^1^	Total	Male	Female	*P*-val.^1^	Total	Male	Female	*P*-val^1^	
**Sex**	38	19 (50.0)	19 (50.0)		55	31 (56.4)	24 (43.6)		138	74 (53.6)	64 (46.4)		0.833
**Age (mean ± SD, years)**	52.74±11.93	50.37±12.23	55.11±11.87	0.232	63.02±11.20	61.94±9.74	64.42±12.71	0.425	66.57±10.94	66.45±9.65	66.70±12.27	0.891	**<0.001**
≥ 65	6 (15.8)	2 (10.5)	4 (21.1)	0.660	24 (43.6)	9 (29.0)	15 (62.5)	**0.016**	84 (60.9)	43 (58.1)	41 (64.1)	0.490	**<0.001**
< 65	32 (84.2)	17 (89.5)	15 (78.9)		31 (56.4)	22 (70.9)	9 (37.5)		54 (39.1)	31 (41.9)	23 (35.9)		
**BMI (mean ± SD, Kg/m** ^**2**^ **)**	23.57±3.02	24.88±2.04	22.26±3.26	**0.006**	23.23±3.23	23.68±3.34	22.65±2.96	0.248	23.70±3.84	24.56±2.94	22.71±4.47	**0.004**	0.713
Obese (≥ 25)	11 (29.0)	8 (42.1)	3 (15.8)	0.151	12 (21.8)	8 (25.8)	4 (16.7)	0.519	45 (32.6)	29 (39.2)	16 (25.0)	0.101	0.331
Underweight (< 18.5)	1 (2.6)	0 (0)	1 (5.3)	1.000	3 (5.5)	1 (3.2)	2 (8.3)	0.575	8 (5.8)	1 (1.4)	7 (10.9)	**0.025**	0.735
**Current/ex-smoker**	13 (34.2)	12 (63.2)	1 (5.3)	**<0.001**	24 (43.6)	23 (74.2)	1 (4.2)	**<0.001**	53 (38.4)	49 (66.2)	4 (6.3)	**<0.001**	0.643
**Alcohol drinker**	22 (57.9)	16 (84.2)	6 (31.6)	**0.003**	29 (52.7)	23 (74.2)	6 (25.0)	**<0.001**	75 (54.3)	52 (70.3)	23 (35.9)	**<0.001**	0.884
**Location**				0.495				0.268				**<0.001**	**0.026**
Proximal	13 (34.2)	5 (26.3)	8 (42.1)		33 (60.0)	21 (67.7)	12 (50.0)		58 (42.0)	20 (27.0)	38 (59.4)		
Distal	25 (65.8)	14 (73.7)	11 (57.9)		22 (40.0)	10 (32.3)	12 (50.0)		80 (58.0)	54 (73.0)	26 (40.6)		

Numbers in parentheses are percentages. Values presented in bold indicate statistically significant differences.

^1^ and ^2^ indicate statistical significance between sex within each group and among three groups, respectively. SD, Standard deviation; BMI, Body mass index.

Each group was further categorized into two subgroups according to sex, and univariate analysis was performed to identify associated risk factors (Table 1). Regarding lesion-related factors, CRCs were more frequently found in the proximal colon among females, whereas males had a higher incidence of CRCs in the distal colon (*P* < 0.001). Furthermore, males aged < 65 years had a higher prevalence of colorectal adenomas (70.9%) compared to older males (29.0%), while older females had a higher proportion of colorectal adenomas than younger females (62.5% and 37.5%, respectively; *P* = 0.016).

### Association between polyp subtypes and PD-L1 expression in colorectal adenomas

Among the 55 patients with colorectal adenomas, 11 patients (20%, including six SSL, four TSA, and one unclassified serrated adenoma) had serrated lesions/polyps and 44 patients (80%, including 37 tubular and 7 tubulovillous adenomas) had conventional adenomas (Table 2). The distribution profile of colorectal adenomas according to subtype classification did not differ between sexes. Serrated lesions/polyps showed a tendency towards positive PD-L1 expression at CPS ≥ 1 (OR 4.24, 95% CI 0.98–18.22, *P* = 0.053), and the incidence of PD-L1 CPS ≥ 5 was significantly higher in patients with serrated lesions/polyps (OR 17.5, 95% CI 3.53–86.8, *P* < 0.001) (Table 2). Likewise, the average PD-L1 CPS was higher in serrated lesions/polyps than in conventional adenomas (5.73 and 1.41, respectively; *P* < 0.001; Fig 2D).

**Table 2:** Association of sex or PD-L1 CPS with colorectal adenoma classification.

	Colorectal adenoma (*N* = 55), *n* (%)												
	Sex			CPS									
	Male (*n* = 31)	Female (*n* = 24)	*P*-val.	CPS < 1 (*n* = 30)	CPS ≥ 1 (*n* = 25)	OR	95% CI	*P*-val.	CPS < 5 (*n* = 44)	CPS ≥ 5 (*n* = 11)	OR	95% CI	*P*-val.
**Serrated lesions/polyps**	6 (54.5)	5 (45.5)	1.000	3 (27.3)	8 (72.7)	4.24	0.98–18.22	0.053	4 (36.4)	7 (63.6)	17.50	3.53–86.8	**< 0.001**
SSL	3	3		1	5				2	4			
TSA	2	2		2	2				2	2			
Serrated adenoma, unclassified	1	0		0	1				0	1			
**Conventional adenomas**	25 (56.8)	19 (43.2)		27 (61.4)	17 (38.6)	1.00			40 (90.9)	4 (9.1)	1.00	-	-
Tubular adenoma	22	15		25	12				34	3			
Tubulovillous adenoma	3	4		2	5				6	1			

Numbers in parentheses are percentages. Values presented in bold indicate statistically significant differences.

CPS, Combined positive score; SSL, Sessile serrated lesions; TSA, Traditional serrated adenomas; OR, Odds ratio; CI, Confidential interval.

### The overall distribution of PD-L1 expression and its association with sex

In total, 14 controls (36.8%), 25 patients with colorectal adenomas (45.5%), and 104 patients with CRC (75.4%) were positive for PD-L1 expression when the CPS cut-off value was set at 1. When the CPS cutoff value was set at 5, three controls (7.9%), 11 patients with colorectal adenomas (20%), and 67 patients with CRC (48.6%) were positive for PD-L1 expression (S1 Table). PD-L1 CPS levels were significantly higher in CRCs than in control and colorectal adenomas (*P* = 0.01 and *P* = 0.004, respectively; Fig 2E), whereas no difference was observed in PD-L1 CPS between the control and adenomas (*P* = 0.758; Fig 2E). Furthermore, during analysis of the relationship between sex and PD-L1 expression, at PD-L1 CPS cutoff of 1 or 5, no significant correlation was observed between sex and PD-L1 expression within the groups, regardless of histopathological diagnosis (S1 Table).

### Comparison of sex and PD-L1 CPS in colorectal carcinomas among various clinicopathological parameters

The results of the univariate analysis of clinicopathological features associated with sex or PD-L1 CPS (cutoff of ≥ 1 or ≥ 5 as positive) are summarized in S2 Table. Lower PD-L1 expression was correlated with an advanced T stage, pathological T (pT) category 4. The percentage of patients with higher PD-L1 CPS (CPS ≥ 5) was significantly lower in pT4 than in pT*is* through pT3 in patients with CRCs (29.2% and 52.6%, respectively, *P* = 0.037; Table 3). No relationship was found between sex, PD-L1 CPS level and other clinicopathological variables (S2 Table).

**Table 3:** PD-L1 CPS according to tumor invasion depth of CRC.

	Colorectal carcinoma (*N* = 138)		
	CPS < 5	CPS ≥ 5	*P*-val.
**Tumor invasion depth, *n* (%)**			**0.037** ^**1**^
pT*is* through pT3 (*n* = 114)	54 (47.4)	60 (52.6)	
pT4 (*n* = 24)	17 (70.8)	7 (29.2)	

Numbers in parentheses are percentages. Values presented in bold indicate statistically significant differences. ^1^Linear-by-linear association chi-square test. pT, Pathological T; *is*, *In situ;* CPS, Combined positive score.

### Factors associated with MMR/MSI status, EGFR, and PD-L1 expression in CRC

Among the 138 patients with CRC, MMR/MSI status was determined by both IHC and PCR in 96 patients (69.6%), IHC alone in 39 patients (28.3%), and PCR alone in 1 patient (0.7%). The results of the univariate analysis of molecular markers, dMMR/MSI-high, EGFR, and PD-L1, related to either sex or tumor location, are shown in Table 4. Sex was significantly associated with the MMR/MSI status and EGFR expression; the incidence of dMMR/MSI-high was prominent in females compared to males (15.6% and 2.7%, respectively, *P =* 0.012), and the overexpression of EGFR was positively associated with female patients compared to males (42.2% and 18.9%, respectively, *P =* 0.006) (Table 4).

**Table 4:** Univariate analysis of sex or tumor location on dMMR/MSI, EGFR, and PD-L1 CPS in patients with CRC.

	Colorectal carcinomas (*N* = 138), *n* (%)								
	dMMR/MSI-high			EGFR			CPS		
	No	Yes	*P*-val.	Low	High	*P*-val.	< 1	≥ 1	*P*-val.
**Sex**			**0.012**			**0.006**			0.484
Female	53 (82.8)	10 (15.6)		36 (56.3)	27 (42.2)		14 (21.9)	48 (78.1)	
Male	71 (95.9)	2 (2.7)		59 (79.7)	14 (18.9)		20 (27.0)	54 (73.0)	
**Location**			0.164			0.132			0.278
Proximal	49 (84.5)	8 (13.8)		35 (60.3)	22 (37.9)		17 (29.3)	41 (70.7)	
Distal	75 (93.8)	4 (7.0)		60 (75.0)	19 (23.8)		17 (21.3)	63 (78.8)	

Numbers in parentheses are percentages. Values presented in bold indicate statistically significant differences. dMMR, Deficient mismatch repair; MSI, Microsatellite instability; EGFR, Epidermal growth factor receptor; CPS, Combined positive score.

Multivariate logistic regression analysis was performed to identify factors associated with MMR/MSI status, EGFR and PD-L1 expression in patients with CRC, respectively (Table 5). The covariates presenting significant sex differences from Table 1 were included in the multivariate analysis. Due to the interaction between sex and tumor location in CRC, analysis for each sex was further stratified by tumor location. Females with proximal CRC showed a significant association with dMMR/MSI-high and high EGFR expression (OR 14.93, 95% CI 1.27–176.05, *P* = 0.032; and OR 4.17, 95% CI 1.28–13.56, *P* = 0.017, respectively), while PD-L1 expression was inversely correlated to males with proximal CRC with a CPS cutoff of 1 (OR 0.28, 95% CI 0.09–0.91, *P* = 0.034) (Table 5).

**Table 5:** Multivariate logistic regression analysis on factors associated with dMMR/MSI, EGFR, and PD-L1 CPS in 138 Patients with CRC.

	Colorectal carcinoma (*N* = 138)											
	dMMR/MSI-high				EGFR high				CPS ≥ 1			
	*n* (%)	OR	95% CI	*P*-val.	*n* (%)	OR	95% CI	*P*-val.	*n* (%)	OR	95% CI	*P*-val.
**Sex/Location**												
Female/Proximal (*n* = 38)	7 (18.4)	14.93	1.27–176.05	**0.032**	19 (50.0)	4.17	1.28–13.56	**0.017**	30 (78.9)	0.44	0.12–1.70	0.236
Female/Distal (*n* = 26)	3 (11.5)	9.98	0.70–142.41	0.090	8 (30.8)	1.50	0.41–5.46	0.539	20 (76.9)	0.32	0.07–1.38	0.126
Male/Proximal (*n* = 20)	1 (5.0)	3.18	0.18–55.26	0.428	3 (15.0)	0.69	0.17–2.84	0.607	11 (55.0)	0.28	0.09–0.91	**0.034**
Male/Distal (*n* = 54)	1 (1.9)	1.00	-	-	11 (20.4)	1.00	-	-	43 (79.6)	1.00	-	-
**Age (years)**												
≥ 65 (*n* = 84)	6 (7.1)	0.53	0.13–2.13	0.373	24 (28.6)	0.68	0.29–1.57	0.365	61 (72.6)	0.60	0.25–1.48	0.270
< 65 (*n* = 54)	6 (11.1)	1.00	-	-	17 (31.5)	1.00	-	-	43 (79.6)	1.00	-	-
**Current/ex-smoker** **(*n* = 14)**	1 (7.1)	0.74	0.10–5.49	0.767	3 (21.4)	1.06	0.36–3.12	0.919	9 (64.3)	0.33	0.11–1.03	0.057
**Alcohol drinker** **(*n* = 75)**	8 (10.7)	4.27	0.92–19.73	0.063	19 (25.3)	0.81	0.34–1.90	0.623	54 (72.0)	0.66	0.26–1.68	0.384
**BMI (Kg/m** ^**2**^ **)**												
Obese (≥ 25) (*n* = 45)	2 (4.4)	0.59	0.11–3.21	0.537	12 (26.7)	1.06	0.44–2.53	0.898	34 (75.6)	1.17	0.47–2.92	0.731
Underweight (< 18.5) (*n* = 8)	2 (25.0)	2.75	0.36–21.07	0.331	4 (50.0)	1.34	0.27–6.65	0.722	7 (87.5)	1.59	0.16–15.41	0.690

Numbers in parentheses are percentages. Values presented in bold indicate statistically significant differences. dMMR, Deficient mismatch repair; MSI, Microsatellite instability; EGFR, Epidermal growth factor receptor; CPS, Combined positive score; CRC, Colorectal cancer; OR, Odds ratio; CI, Confidential interval; BMI, Body mass index.

No correlation was found between PD-L1 CPS level and the distribution of MMR/MSI status or EGFR expression in CRCs (S3 Table). In the analysis for survival outcomes in patients with CRC using the Kaplan-Meier method, both overall and colorectal cancer-specific survival was not affected by MMR/MSI status, EGFR expression, and PD-L1 CPS level (S2 Fig), which could be originated from the small number of patients who expired during follow-up.

## Discussion

MMR/MSI status and EGFR expression showed sex-specific molecular features when stratified according to tumor location. In the multivariate analysis, females with proximal CRC had a higher frequency of dMMR/MSI-high and a higher EGFR expression. In addition, PD-L1 showed a significantly reduced positivity (CPS ≥ 1) in males with proximal CRC, and patients with CRC who had a higher PD-L1 expression (CPS ≥ 5) were significantly less frequent in the advanced T stage. Furthermore, serrated lesions/polyps showed a markedly higher expression of PD-L1 (CPS ≥ 5) compared with conventional adenomas. To the best of our knowledge, the present study is the first in which relationship of sex and PD-L1 expression was analyzed based on tumor types, including colorectal adenomas.

In the past few decades, immunotherapy has achieved a certain degree of success in cancer treatment, allowing rapid incorporation of ICIs into clinical practice. The FDA has approved nivolumab and pembrolizumab, PD-1 blocking antibodies, for dMMR/MSI-high metastatic CRC regardless of PD-L1 expression [33–35]. It has been shown that colorectal tumor cells rarely expressed PD-L1, and the expression of PD-L1 was rather to tumor-infiltrating lymphocytes (TILs) [36], which might be due to the heterogeneity of PD-L1 expression [37]. In fact, several clinical trials have reported that the level of PD-L1 expression in metastatic CRC was not significantly associated with response to ICI treatment [33,38]. Meanwhile, recent *in vitro* and *in vivo* studies have revealed that PD-L1 expression was positively correlated with tumor growth in CRC, and tumor-intrinsic signaling of PD-L1 regulated tumorigenesis beyond immune evasion in a variety of tumor cell types including CRC [11,39,40]. In our study, PD-L1 expression was significantly increased in serrated lesions/polyps including sessile serrated lesions, pre-malignant lesions associated with the serrated neoplasia pathway in colorectal carcinogenesis, compared with conventional adenomas. On the other hand, CRCs with advanced T stage showed lower PD-L1 expression. The elevated PD-L1 expression in serrated lesions/polyps in the present study is in line with a previous report where a stepwise increase in PD-L1 expression was shown in sessile serrated lesions as cytological dysplasia progressed, and the upregulation of PD-L1 in patients with sessile serrated lesions was detected prior to the development of dMMR [14]. Similarly, other studies also demonstrated that the incidence of PD-L1 positivity was decreased in the advanced TNM stage in CRC [41,42] and in the head and neck squamous cell carcinomas as well [43]. Taken together, these findings may provide potential evidence for further investigations on the extended use of anti-PD-L1 immunotherapy outside the metastatic setting as adjuvant or neoadjuvant treatment in patients with early-stage CRC.

The efficacy of ICIs has been shown to be affected by sex. Female patients with cancer showed less benefit from immunotherapy when used as a single agent for lung cancer and melanoma [44]. Similarly, both anti-PD-1 and PD-L1 therapies exhibit better survival benefits in male patients with CRC [44]. Despite a wide body of literature suggesting a distinct sexual dimorphism in CRC, there are few studies regarding the influence of sex on immunotherapy for CRC. Previously we reported *in vivo* study demonstrating that co-treatment with 17β-estradiol and an anti-PD-L1 antibody significantly reduced PD-L1 expression as well as MC38 colon tumor growth in male mice [40]. Thus, we further investigated not only PD-L1 but also MMR/MSI status in humans, to understand the association of these molecular biomarkers with sex. Although estrogen exhibited an inhibitory effect on PD-L1 expression in CRC in mice [39], the expression of PD-L1 in CRC did not differ between the sexes in this study, similar to a previous report [45]. Likewise, in the present study, when each sex and tumor location was set as an independent variable in a multivariate analysis, neither sex nor tumor location had a correlation with MMR/MSI status and EGFR expression, possibly due to the strong interaction effect between sex and tumor location in CRC [15]. Considering the substantial influence of both tumor location and sex in CRC, we speculated that stratifying the study subjects to either tumor location or sex alone may result in inconclusive findings, and both sex and tumor location should be taken together to evaluate location-dependent sex differences in patients with CRC. Additionally, sex and tumor location cannot be considered as separate variables in the actual clinical settings as the patients are either female or male and with proximal or distal primary tumors at the same time. Interestingly, when tumor location was subsequently combined with sex in the present study, within the male patients, patients with proximal tumor location had a significantly lower incidence of positive PD-L1 expression compared to patients with distal location. Furthermore, females with proximal CRC showed a markedly higher incidence of dMMR/MSI-high status, while males did not exert location dependency in MMR/MSI status, indicating that sex and tumor location should be taken into consideration in selecting patients with CRC for biomarker analysis.

EGFR is one of the most important therapeutic targets for CRC treatment. Functional polymorphisms of *EGFR* had been associated with sex differences in CRC survival [46], and sex affects the mutational frequency of downstream effectors of EGFR in patients with CRC [47]. In the present study, we observed that female patients prominently had high EGFR expression, consistent with the previous reports suggesting that EGFR is regulated by estrogen [48,49]. Moreover, bidirectional signaling between estrogen receptor and EGFR has been demonstrated in many cancer types [50].

Anti-EGFR therapies have demonstrated improved efficacy and survival benefits when combined with chemotherapy, and these combinations of chemotherapy plus anti-EGFR mAb or anti-vascular endothelial growth factor mAb are widely used in the current clinical settings as first-line treatment options for advanced CRC [51]. Notably, several studies identified that distal CRCs having lower EGFR expression showed better prognosis to anti-EGFR therapies compared to those proximal [52,53]. Likewise, in an attempt to enhance the response to immunotherapy in CRC, several clinical trials are currently investigating the therapeutic effects of drug combinations, such as ICIs with chemotherapy and/or targeted agents [9]. In the present study, we observed that the frequency of dMMR/MSI-high or high EGFR expression was independent of PD-L1 expression at either CPS ≥ 1 or CPS ≥ 5 in CRC. However, the subgroups of patients with CRC stratified by sex and subsequent tumor location had distinctive molecular patterns of PD-L1 expression, MMR/MSI status, and EGFR expression, which could have a potential predictive role in patient screening for combination therapies.

Our study has several limitations. First, the PD-L1 cutoff of either CPS ≥ 1 or CPS ≥ 5 was used for the analysis because no consensus has been reached for the detection modality and scoring system of PD-L1 in CRC. Thus, we set two different cutoff points for a PD-L1 positive status as CPS ≥ 1 and a higher PD-L1 expression as CPS ≥ 5 to overcome the discrepancies in CPS cutoff value in CRC. Second, the number of enrolled patients with colorectal adenomas was small, possibly due to rather strict exclusion criteria, recruitment process, and a relatively lower prevalence of serrated lesions/polyps, which may lead to low statistical power; therefore, the influence of sex in colorectal adenomas including serrated lesions/polyps should be validated in a larger cohort. Further, it was difficult to estimate the sample size based on a single variable since this study focused on multiple markers. Besides, the cutoff value and prevalence of PD-L1 positivity in the population with CRC are controversial. Therefore, the sample size calculation was based on the previously reported incidence of MSI-high in CRCs [31]. A follow-up study is underway to evaluate the influence of sex and tumor location on the mechanism regarding immune-related carcinogenesis in a larger sample size.

In conclusion, sex and tumor location significantly influenced key molecular features such as PD-L1, MMR/MSI status and EGFR expression in CRC, suggesting that understanding sex differences together with tumor location may provide a clue to the possible development of future personalized therapeutic strategies in patients with CRC. Additional research in a larger number of colorectal adenoma cohort is needed to evaluate the molecular differences between sexes and their interaction with PD-L1 expression in colorectal adenomas.

## Supporting information

S1 Fig Representative immunohistochemical staining of EGFR and MMR in CRC tissue. (A) EGFR high-expression; (F) EGFR low-expression. Proficient MMR (pMMR) showing the expression of all four MMR proteins, including MLH1 (B), MSH2 (C), MSH6 (D), and PMS2 (E); Deficient MMR (dMMR) showing loss of MLH1 (G) and PMS2 (J) expression, and retained expression of MSH2 (H) and MSH 6 (I) in tumor cells. magnification, x200. CRC, Colorectal cancer; EGFR, Epidermal growth factor receptor; MMR, Mismatch repair protein.
(TIF)

S2 Fig Kaplan-Meier survival curves showing the correlation between molecular markers and overall survival or colorectal cancer-specific survival. (A-C) Kaplan-Meier curves for overall survival of colorectal cancer (CRC) patients according to PD-L1 CPS (A), MMR/MSI status (B), and EGFR expression (C). (D-F) Kaplan-Meier curves for CRC-specific survival of the patients according to PD-L1 CPS (D), MMR/MSI status (E), and EGFR expression (F). CPS, Combined positive score; CRC, Colorectal cancer; pMMR, Proficient mismatch repair; dMMR, Deficient mismatch repair; MSS, Microsatellite Stable; MSI, Microsatellite instability; EGFR, Epidermal growth factor receptor.
(TIF)

S1 Table PD-L1 CPS in control, colorectal adenoma, and CRC. (TIF)

S2 Table Pathological features of CRC and its association with sex and PD-L1 CPS. (TIF)

S3 Table Interrelation among MMR/MSI Status, EGFR expression and PD-L1 CPS levels. (TIF)

S4 Table Association of tumor location and colorectal adenoma subtypes. (TIF)
