# Supplementary figures and images for: Influence of location-dependent sex difference on PD-L1, MMR/MSI, and EGFR in colorectal carcinogenesis
Source: PLoS One. 2023 Feb 21;18(2):e0282017. doi: 10.1371/journal.pone.0282017 (PMC9942979; doi:10.1371/journal.pone.0282017)

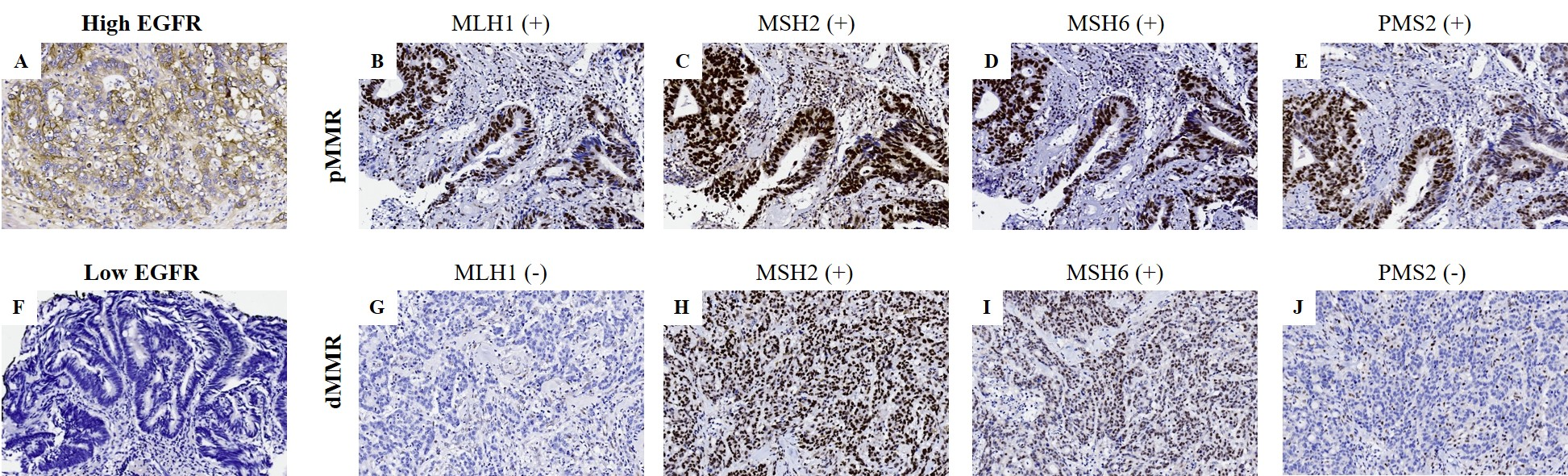

Supplement: S1 Fig — (A) EGFR high-expression; (F) EGFR low-expression. Proficient MMR (pMMR) showing the expression of all four MMR proteins, including MLH1 (B), MSH2 (C), MSH6 (D), and PMS2 (E); Deficient MMR (dMMR) showing loss of MLH1 (G) and PMS2 (J) expression, and retained expression of MSH2 (H) and MSH 6 (I) in tumor cells. magnification, x200. CRC, Colorectal cancer; EGFR, Epidermal growth factor receptor; MMR, Mismatch repair protein. (TIF) [file pone.0282017.s001.tif]

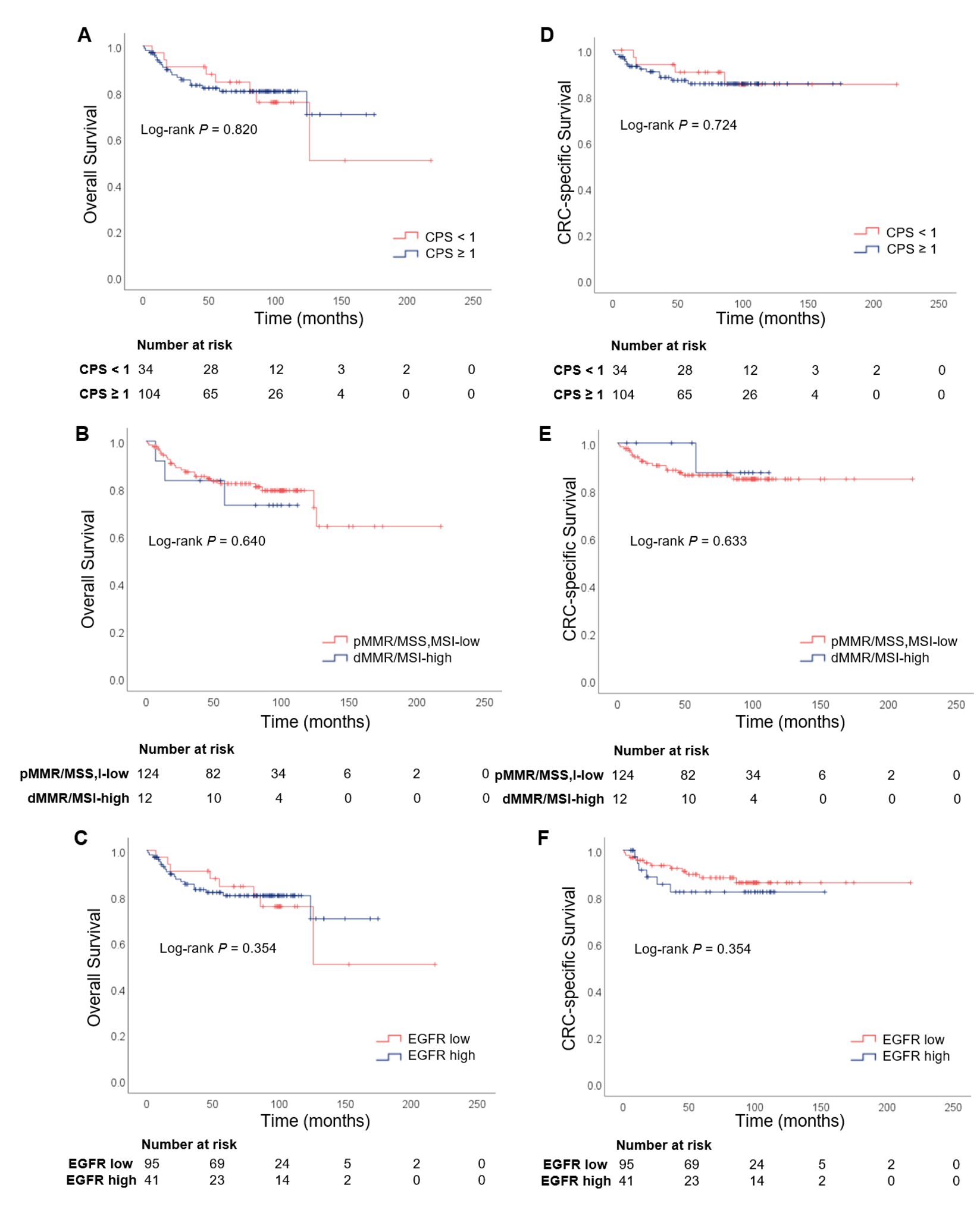

Supplement: S2 Fig — (A-C) Kaplan-Meier curves for overall survival of colorectal cancer (CRC) patients according to PD-L1 CPS (A), MMR/MSI status (B), and EGFR expression (C). (D-F) Kaplan-Meier curves for CRC-specific survival of the patients according to PD-L1 CPS (D), MMR/MSI status (E), and EGFR expression (F). CPS, Combined positive score; CRC, Colorectal cancer; pMMR, Proficient mismatch repair; dMMR, Deficient mismatch repair; MSS, Microsatellite Stable; MSI, Microsatellite instability; EGFR, Epidermal growth factor receptor. (TIF) [file pone.0282017.s002.tif]

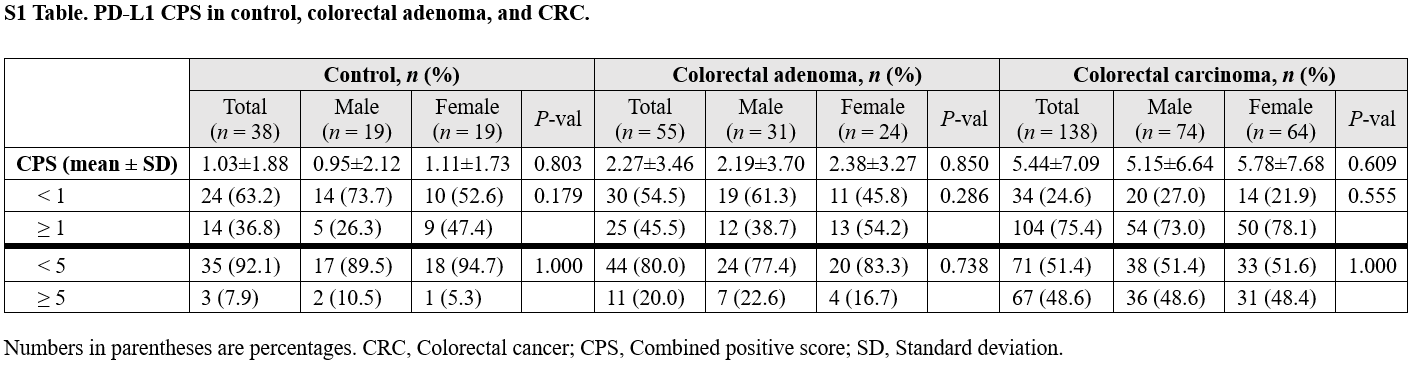

Supplement: S1 Table — (TIF) [file pone.0282017.s003.tif]

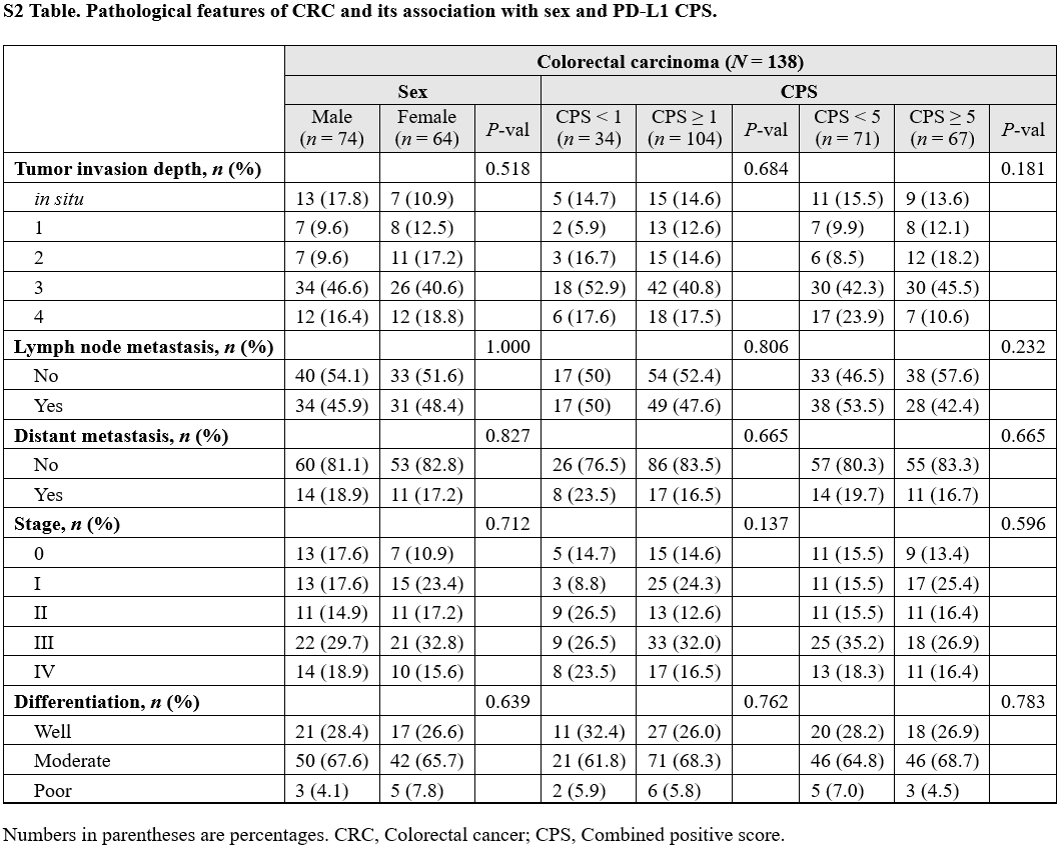

Supplement: S2 Table — (TIF) [file pone.0282017.s004.tif]

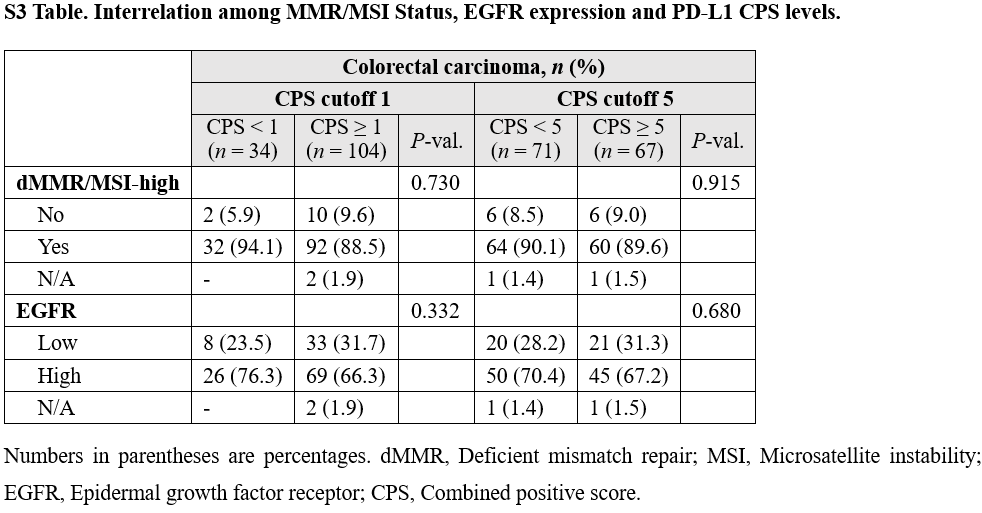

Supplement: S3 Table — (TIF) [file pone.0282017.s005.tif]

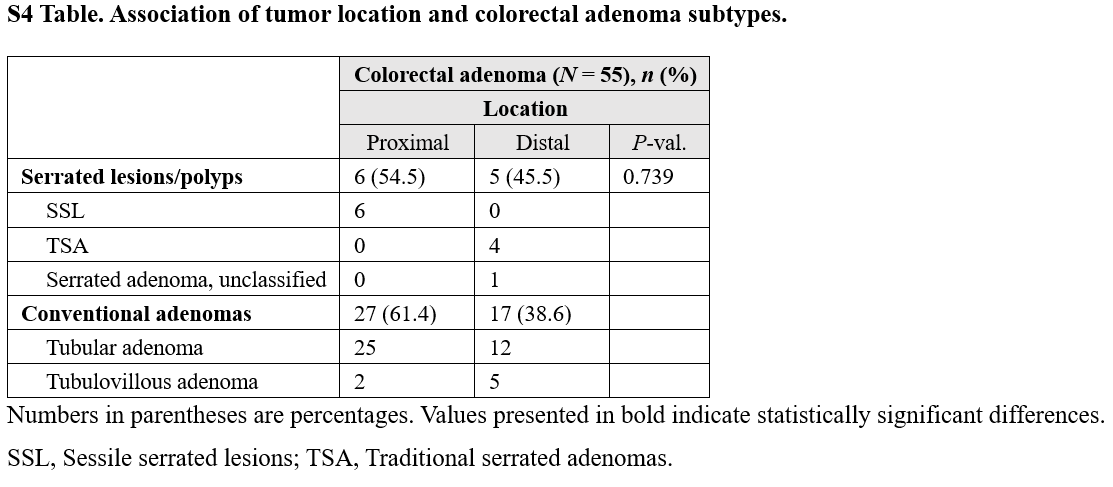

Supplement: S4 Table — (TIF) [file pone.0282017.s006.tif]
